# Attenuating Effect of a Polyphenol Ellagic Acid on Ovarian Aging by Inhibiting the Ferroptosis Pathway in Low-Yield Laying Chickens

**DOI:** 10.3390/antiox14050614

**Published:** 2025-05-21

**Authors:** Qiongyu Yuan, Xinyu Wang, Yingyu Xiao, Zhaoyu Yang, Xiangyu Cai, Wanyue Gao, Yuling Mi, Caiqiao Zhang

**Affiliations:** Department of Veterinary Medicine, College of Animal Sciences, Zhejiang University, Hangzhou 310058, China; qiongyu@zju.edu.cn (Q.Y.); 22217098@zju.edu.cn (X.W.); 22317125@zju.edu.cn (Y.X.); 12117050@zju.edu.cn (Z.Y.); 22317105@zju.edu.cn (X.C.); 22317120@zju.edu.cn (W.G.)

**Keywords:** ovarian aging, ellagic acid, oxidative stress, ferroptosis, chicken

## Abstract

Aging leads to ovarian degeneration in poultry, reducing egg production and quality. Ellagic acid (EA), a natural plant-derived compound, may help delay ovarian aging, though its precise mechanisms remain unclear. This study investigated the effects of EA on ovarian aging of low-yield laying chickens and explored its underlying mechanism. EA supplementation (100 and 500 mg/kg) significantly increased ovarian weight as well as the number and proportion of small yellow follicles in aging chickens. EA administration elevated serum antioxidant levels and upregulated the expression of glutathione peroxidase 4 (GPX4) expression to reduce oxidative stress. Importantly, EA treatment suppressed the mRNA and protein expression of ferroptosis markers transferrin receptor protein 1 (TFRC) and solute carrier family 7 member 11 (SLC7A11), increased Proliferating Cell Nuclear Antigen (PCNA) expression, and alleviated G1 phase arrest in granulosa cells (GCs), promoting cell proliferation, which improves egg quality and production. Furthermore, in vitro experiments demonstrated that EA treatment decreased reactive oxygen species production, improved mitochondrial function, inhibited ferroptosis, and attenuated GCs aging. In conclusion, this study reveals the critical role of ferroptosis in chicken ovarian aging and suggests that EA may provide a promising approach for delaying ovarian aging and enhancing productivity in low-yield poultry.

## 1. Introduction

In female animals, ovarian aging precedes the decline of other organs, with diminished ovarian reserve leading to reduced fertility [1]. In poultry, egg-laying capacity is closely linked to ovarian health. The ovary contains small white follicles (SWFs), large white follicles (LWFs), small yellow follicles (SYFs), large yellow follicles (LYFs), five to six preovulatory follicles (F1–F6), and several postovulatory follicles (POFs) that lack oocytes. Granulosa cells (GCs) and theca cells (TCs) play critical roles in follicular development [2]. As chickens age, particularly beyond 580 days, the quantity and quality of oocytes decline significantly, accompanied by a sharp reduction in egg production and egg quality. This decline results in substantial economic losses to the poultry industry [3].

The mechanisms of ovarian aging involve various factors, such as gene mutations, DNA damage, and mitochondrial dysfunction. Other contributing factors include apoptosis [4,5] (particularly of oocytes and GCs), oxidative stress [6,7], cellular senescence [8], and lipid metabolism [9]. Oxidative stress arises from an imbalance between reactive oxygen species (ROS) production and the antioxidant defense system [10]. Under normal conditions, ROS act as signaling molecules in folliculogenesis and ovulation [11]. However, with increasing age, ROS levels in the ovary rise significantly, leading to oxidative damage that impairs the DNA and cellular structures of oocytes, ultimately reducing oocyte quality and follicular reserve. The accumulation of oxidative stress renders the female reproductive system [12] increasingly vulnerable to aging and oxidative damage [13]. Thus, investigating the role of oxidative stress in ovarian aging is of critical importance.

Ferroptosis represents a newly identified modality of cell death distinct from the traditional mechanisms of necrosis, apoptosis, and autophagy [14]. Its discovery has expanded our understanding of how cells undergo regulated death. Distinct from apoptotic mechanisms, ferroptosis is characterized as an iron-dependent, regulated necrotic process driven by extensive lipid peroxidation-induced membrane damage [15]. Ferroptosis is driven by three primary pathways. First, intracellular iron ions are imported via transferrin receptors and metal transporters (such as SLC39A14), followed by the generation of ROS through endosomal iron reductase and the Fenton reaction, leading to lipid peroxidation and cellular damage [16]. Second, the inactivation of glutathione peroxidase 4 (GPX4) and the depletion of its cofactor glutathione (GSH), which leads to the accumulation of lipid peroxides by either inhibiting cysteine uptake or directly inhibiting GPX4, ultimately resulting in cell death [17]. Third, the peroxidation of polyunsaturated fatty acid (PUFA) phospholipids, where acyl-CoA synthetase long-chain family member 4 catalyzes the formation of PUFA-CoA, and lipoxygenases promote PUFA phospholipid peroxidation, causing cell membrane damage [18]. The core mechanism involves an iron-dependent accumulation of excessive ROS and a weakened GPX4-mediated clearance, leading to a dysregulation of the ROS generation and degradation homeostasis. In all relevant pathways, the accumulation of iron-dependent lipid ROS is closely associated with ferroptosis. During aging, intracellular iron overload persists, and high concentrations of iron generate excessive ROS through the Fenton reaction. This results in oxidative stress that overwhelms the cell’s normal antioxidant defense mechanisms due to the excessive iron [19]. As the cell’s ability to handle oxidative stress continues to decline and eventually becomes exhausted, ferroptosis accelerates the aging process of the cell.

Existing literature suggests that ferroptosis may exert a regulatory role in the ovarian aging process in chickens [20]. In recent years, natural plant extracts have gained attention in research for their potential anti-aging and antioxidant properties, particularly in mitigating oxidative stress and cellular aging. Notable examples include resveratrol [21], curcumin [22], proanthocyanidins [23], naringin [24], epimedium, quercetin, ginsenosides, magnolol [25], matrine, and puerarin. Ellagic acid (EA, 2,3,7,8-tetrahydroxychromeno [5,4,3-cde] chromene-5,10-dione; C14H6O8) is a natural polyphenolic compound found in fruits such as berries and pomegranates [26]. EA has demonstrated antioxidant [27], anti-inflammatory, antifibrotic, and anti-aging [28] effects in various disease models. It regulates oxidative stress by scavenging free radicals, enhancing catalase (CAT) and superoxide dismutase (SOD) activities, maintaining glutathione (GSH) levels, inhibiting lipid peroxidation, and reducing ROS production. These actions help protect cells from age-related oxidative damage.

However, research on EA’s role in mitigating ovarian aging in chickens is limited. It has been shown that EA delays liver fibrosis by inducing ferritin-dependent ferroptosis and disrupting SNARE complex formation [29]. Its role in ovarian aging, particularly through ferroptosis inhibition, remains underexplored.

This study evaluated the effect of EA on laying performance of low-yield laying chickens, then investigated the roles of ROS and ferroptosis involved in the action of EA. It aims to investigate EA’s potential in preventing ovarian aging in chickens by inhibiting ferroptosis. We also analyzed the effects of EA on the mRNA and protein expression of the cell proliferation-related gene PCNA and assessed how ferroptosis impacts the cell cycle of GCs. This research aims to provide new theoretical insights and practical guidance for enhancing egg production and poultry health.

## 2. Materials and Methods

### 2.1. Experimental Animals

Hy-Line White chickens were sourced from Huajie Poultry Company (Hangzhou, China) and managed under standard practices. A total of 36 chickens, aged 580 days (D580), with body weights ranging between 1.5 and 2.0 kg, were randomly divided into three groups: EA low-dose feeding group (100 mg/kg), EA high-dose feeding group (500 mg/kg), and Control group, fed with a basal diet. Each group consisted of 12 chickens, housed in nine cages with four chickens per cage. The experiment was approved by the Zhejiang University Animal Ethics Committee (ZJU20220085).

All chickens were continuously fed for more than 1 month in a commercial laying hen facility with standard conditions, including a temperature of 20–24 °C, a humidity of 50–70%, and a light cycle of 16 h of light and 8 h of darkness.

### 2.2. Tissue Collection and Culture

Liver, ovarian tissues, and different-stage follicles (SWFs, LWFs, SYFs, LYFs) were collected from D280 and D580 chickens, quickly cryopreserved or fixed for experiments. SWFs were isolated, washed with phosphate-buffered saline (PBS), and cultured in DMEM with 5% fetal bovine serum (FBS, Hyclone, Tauranga, New Zealand) plus 1% penicillin/streptomycin (Invitrogen, Carlsbad, CA, USA) at 38.5 °C, 5% CO_2_ for 24 h. EA (Shanghai Yuanye Bio-Technology Co., Ltd., Shanghai, China) concentration and culture time were optimized. Some samples underwent BrdU incorporation and were fixed for immunofluorescence staining, while others were used for qRT-PCR and Western blot analysis.

### 2.3. Cell Culture

SYFs and hierarchical follicles (F1–F5) were collected from D280 and D580 chickens. The granulosa layer was digested with 1 mg/mL collagenase II (Gibco, Grand Island, NY, USA), filtered, and centrifuged to obtain cell pellets. After washing with PBS, cells were resuspended in F12 medium (Hyclone, Tauranga, New Zealand) with 10% FBS and 1% penicillin/streptomycin, seeded at 1 × 10^5^ cells per well, and cultured at 38.5 °C, 5% CO_2_.

EA and Ferrostatin-1 (Fer-1, ferroptosis inhibitor, MCE, Monmouth Junction, NJ, USA) were tested for optimal concentration and treatment time in an oxidative stress aging model. GCs were divided into four groups: control, 200 μM H_2_O_2_-induced aging, 10 μM Fer-1, and 1 μM EA treatment group.

### 2.4. Cell Viability Assay

Cell viability was assessed using the Cell Counting Kit-8 (CCK-8; Land Biology Technology Co., Ltd., Fuzhou, China). GCs were seeded in 96-well plates in 100 μL and allowed to reach approximately 90% confluency. After treatments for 24 h, 10 μL of CCK-8 reagent was added to each well for 2 h. Absorbance at 450 nm was measured using a microplate reader (Varioskan Flash, Thermo Scientific, Waltham, MA, USA).

### 2.5. Western Blot Analysis

Tissue samples were homogenized in RIPA lysis buffer (P0013B, Beyotime, Shanghai, China) containing 1% protease inhibitor PMSF (Solarbio, Beijing, China). The total protein concentration of the supernatant was determined using the BCA protein assay kit. Protein samples were mixed with loading buffer to achieve a final concentration of 3 μg/μL and denatured at 100 °C for 10 min. A volume of 10 µL of the denatured protein samples was loaded onto a 4–20% SDS-polyacrylamide gel and electrophoresed at a constant voltage of 200 V for 1 h. Subsequently, proteins were transferred onto nitrocellulose membranes (Millipore, Darmstadt, Germany) at 200 mA constant current for 45 min. The membrane was then blocked with 4% skimmed milk powder in Tris-buffered saline with Tween-20 (TBST, pH 7.4) for 2 h at room temperature, followed by overnight incubation at 4 °C with primary antibodies, including β-actin (1:10,000, EM21002), TFRC (1:1000, ET1702-06, HuaBio, Hangzhou, China), PCNA (1:1000, ab29, Abcam, Cambridge, UK), GPX4 (1:1000, A22161), SLC7A11 (1:1000, A2413, Abclonal, Wuhan, China).

After washing three times with TBST, the membrane was incubated with secondary antibodies at room temperature for 1 h. The secondary antibodies included HRP-conjugated goat anti-rabbit IgG (1:50,000, HA1001) or HRP-conjugated goat anti-mouse IgG (1:5000, HA1006, HuaBio, Hangzhou, China). Finally, protein bands were detected using an enhanced chemiluminescence kit (Bio-Rad, Hercules, CA, USA) and ChemScope 3400 Mini system (Clinx, Shanghai, China). Protein expression levels were quantitatively analyzed using ImageJ v2.3.0 software.

### 2.6. RNA Extraction and qRT-PCR

The tissue samples from chickens were rapidly frozen in liquid nitrogen and ground into powder. RNA was extracted using TRIzol reagent (Invitrogen, Carlsbad, CA, USA), and the RNA concentration and purity were determined using a NanoDrop spectrophotometer (A260/A280 ratio).

Following the instruction of the reverse transcription kit (Vazyme, Nanjing, China), 1 µg of RNA template was mixed with reverse transcriptase to synthesize cDNA. According to the SYBR Green qPCR kit instructions (Vazyme, Nanjing, China), the reaction system was prepared, including cDNA template, specific primers, and SYBR Green mix, and then added to a 96-well plate (Table S1). The qRT-PCR was performed with an initial denaturation at 95 °C for 5 min, followed by 40 cycles of 95 °C for 15 s and 60 °C for 1 min. Melting curve analysis was conducted to verify the specificity of amplification, and Ct values were analyzed. The relative expression levels of target genes were calculated using the 2^−ΔΔCt^ method, with housekeeping genes such as *GAPDH* as the normalization references.

### 2.7. Tissue Morphological Observation by H&E Staining

Hematoxylin and eosin (H&E) staining: the tissue was fixed in 4% paraformaldehyde for 24–48 h, dehydrated through a series of ethanol solutions (70–100%), and embedded in paraffin. Sections were cut at 5 μm thickness and stained according to standard H&E staining procedures. Tissue morphology was then observed using an Eclipse 80i microscope (Nikon, Tokyo, Japan).

### 2.8. β-Galactosidase Staining

β-Galactosidase staining was performed on GCs using a β-Galactosidase Staining Kit (G1580, Solarbio, Beijing, China) to assess cellular senescence. Following rinsing with sterile PBS, GCs were fixed with β-Galactosidase fixative at room temperature for 15 min. Subsequently, excess fixative was removed by PBS washing. The β-Galactosidase staining working solution was prepared as per the kit’s instructions, and the samples were incubated with the working solution overnight at 37 °C.

### 2.9. Oil Red O Staining

The frozen sections were left at room temperature for 10 min, then stained with Oil Red O solution (G1015-100ML; Servicebio, Wuhan, China) for 15 min. After staining, the sections were gently washed with distilled water to remove excess dye. Hematoxylin was then used for nuclear staining for approximately 1 min, followed by another wash with distilled water until the sections were clear. Finally, the stained sections were imaged using a microscope, such as the Eclipse 80i (Nikon, Tokyo, Japan).

### 2.10. BrdU Staining

The tissue sections were washed with phosphate-buffered saline PBS, fixed with 10% methanol for 5–10 min, and then treated with 0.1 M hydrochloric acid for acid denaturation before being washed with PBS. The sections were then incubated in a 10 μM BrdU labeling solution at room temperature for 1–2 h, followed by a PBS wash. Next, the sections were incubated with anti-BrdU antibody for 1 h at room temperature or overnight at 4 °C, then stained with appropriate secondary antibodies and DAPI for observation.

### 2.11. Mitochondrial Membrane Potential

The mitochondrial membrane potential (MMP) of GCs was assessed using the JC-1 assay kit (Beyotime, Shanghai, China) following the manufacturer’s instructions. Fluorescence values were measured using a Synergy-4 multi-functional enzyme reader (BioTek, Winooski, VT, USA) at excitation wavelengths of 490 nm and 525 nm, and emission wavelengths of 530 nm and 590 nm. Changes in MMP were analyzed based on the ratio of red to green fluorescence intensity to determine the cellular mitochondrial status.

### 2.12. Detection of ROS

ROS-sensitive probe 2′,7′-dichlorodihydrofluorescein diacetate (DCFH-DA Beyotime, Shanghai, China) was employed to detect the level of intracellular ROS production. Cells in a 12-well plate were washed twice with PBS and then incubated at 37 °C in 0.5 mL of assay solution (1:1000 dilution of DCFH-DA) in serum-free medium for 30 min. Subsequently, excess DCFH-DA was removed by washing with PBS. Hoechst 33342 (HY-15559A; MCE, Princeton, NJ, USA) was used to stain the nuclei of GCs cells. Fluorescence intensity was observed under a fluorescence microscope to determine the intracellular ROS production.

### 2.13. Transmission Electron Microscopy (TEM) Analysis

Tissue samples were collected and rapidly transferred to 2.5% glutaraldehyde, fixed overnight at 4 °C, thoroughly washed with PBS to complete the fixation, and then transferred to a 1% osmium tetroxide solution for an additional 1 h fixation. The samples were dehydrated using a gradient concentration of ethanol solutions (30–95%), with each concentration treated for 15 min followed by treatment with pure ethanol for 20 min, pure acetone for 20 min, and a mixture of Spurr embedding resin and acetone (*v*/*v* = 1/1) for 1 h, and a mixture of Spurr embedding resin and acetone (*v*/*v* = 3/1) for 3 h. Finally, the samples were transferred to pure embedding resin and left overnight at room temperature. The samples were heated overnight at 70 °C for complete embedding, sectioned using a LEICA EM UC7 ultramicrotome, stained with lead citrate solution and 50% ethanol-saturated uranyl acetate for 5–10 min each, and observed directly under a Hitachi H-7650 transmission electron microscope (Hitachi High-Technologies Corporation, Tokyo, Japan).

### 2.14. Serum Biochemical Analysis

Blood samples were collected from the wing vein by puncturing the brachial vein of chickens and collected into anticoagulant tubes. The blood was then centrifuged to separate the serum for analysis. The levels of aspartate aminotransferase (AST), alanine aminotransferase (ALT), glucose (GLU), low-density lipoprotein (LDL), high-density lipoprotein (HDL), triglycerides (TG), and total cholesterol (TC) in the serum were measured using an automatic biochemical analyzer (Bs-300, Mindray, Shenzhen, China).

### 2.15. Biochemical Analysis

According to the instructions provided by the reagent kits (Nanjing Jiancheng Bioengineering Institute, Nanjing, China), the corresponding tissues from the chickens were homogenized, centrifuged, diluted, and pretreated for sample preparation. The following parameters were measured: malondialdehyde (MDA), GSH, CAT, SOD, tissue iron content, total Cholesterol (T-CHO), and TG. The samples were then analyzed using a BioTek Synergy H1 multi-mode microplate reader (BioTek Instruments, Inc., Winooski, VT, USA).

### 2.16. Statistical Analysis

All experimental data were obtained from more than three independent experiments, with six or morebiological replicates and three or more technical replicates. Collected data were analyzed using either an unpaired Student’s *t*-test or one-way/two-way ANOVA, with appropriate post hoc tests conducted using GraphPad Prism 8.0 (GraphPad Software, San Diego, CA, USA) or SPSS 25.0. Data were plotted as means ± SEM for statistical analysis. Relative optical density analysis of Western blots was performed using ImageJ 1.40 (NIH, Bethesda, MD, USA) and normalized. Statistical significance was set at *p* < 0.05 unless otherwise indicated.

## 3. Results

### 3.1. Follicle Distribution in D280 and D580 Chickens

**Figure 1:**
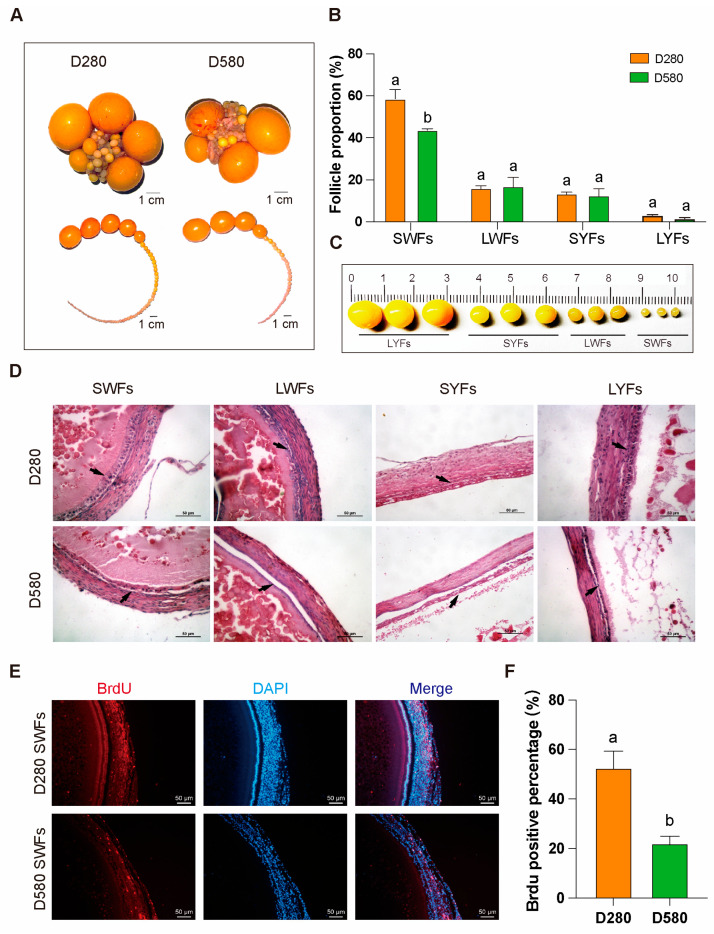
Differences in pre-ovulatory follicles between D280 and D580 chickens. (**A**–**C**) Comparison of the distribution of pre-ovulatory follicles at various stages in D280 and D580 chickens, *n* = 6, scale bar: 1 cm. (**D**) H&E staining of pre-ovulatory follicles at various stages in D280 and D580 chickens. Black arrows point to the granulosa layer of follicles, *n* = 3, scale bar: 50 µm. (**E**,**F**) BrdU staining and analysis of SWFs in D280 and D580 chickens, *n* = 3, scale bar: 50 µm. All data are presented as means ± SEM, and different lowercase letters indicate significant differences (*p* < 0.05).

Ovarian tissues from D280 and D580 were dissected or morphological observation and statistical analysis of pre-ovulatory follicles. Compared to D280, aging chickens exhibited a significantly lower proportion of SWFs (Figure 1A–C). H&E staining further revealed that, in D580, the granulosa and membrane layers of follicles (SWFs, LWFs, SYFs, LYFs) were notably separated, with GCs loosely arranged, in contrast to the more compact structure in D280 (Figure 1D). Additionally, BrdU labeling index was also significantly higher than in D580 SWFs (Figure 1E,F).

### 3.2. Ferroptosis Occurrence in SWFs of D280 and D580 Chickens

**Figure 2:**
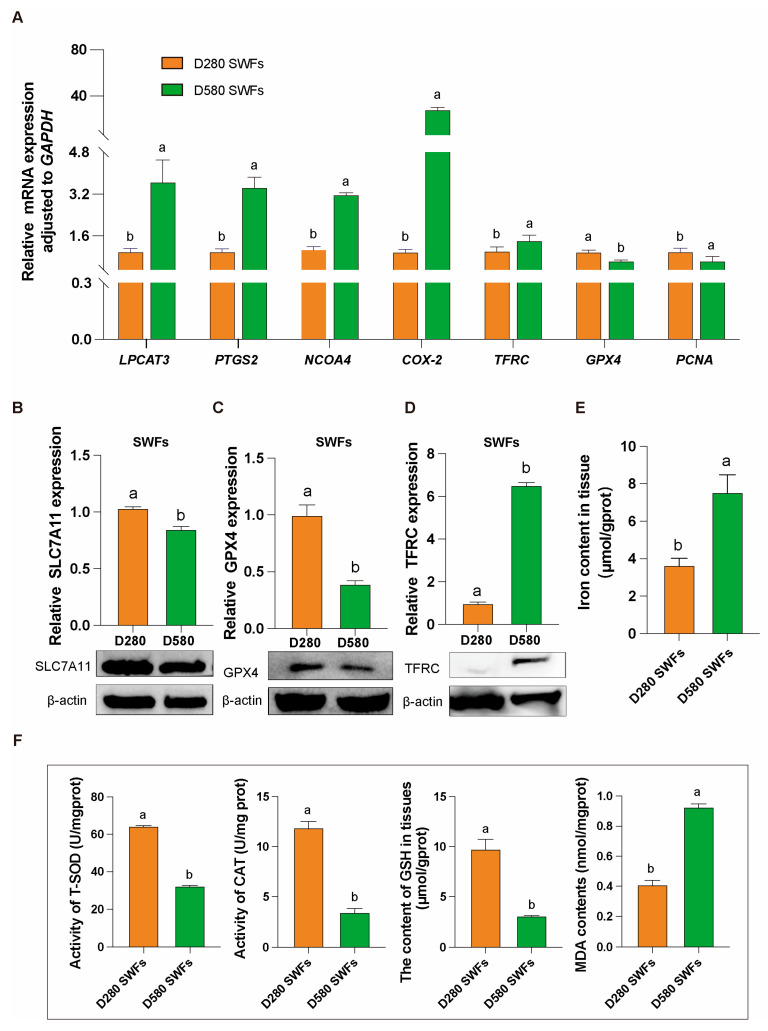
Gene, protein and biochemical analysis in D280 and D580 chickens’ SWFs. (**A**) mRNA expression levels of ferroptosis-related genes (*LPCAT3*, *PTGS2*, *NCOA4*, *COX2*, *TFRC*) and cell proliferation-related gene (*PCNA*) in D280 and D580 SWFs, *n* = 6. (**B**–**D**) Western blot analysis of ferroptosis-related proteins SLC7A11, GPX4, and TFRC in D280 and D580 SWFs, *n* = 6. (**E**,**F**) Determination of iron content, T-SOD, CAT, GSH, and MDA levels in D280 and D580 SWFs, *n* = 6. All data are presented as means ± SEM (*n* ≥ 3), and different lowercase letters indicate significant differences (*p* < 0.05).

Under natural physiological conditions, D280 and D580 chickens were directly dissected to obtain ovarian tissues and isolate SWFs in their entirety. The isolated SWFs underwent protein, gene expression, and biochemical analyses. qRT-PCR results indicated significant upregulation of mRNA expression of genes related to promoting ferroptosis, including *LPCAT3*, *PTGS2*, *NCOA4*, *COX2*, and *TFRC*, in aging chickens’ SWFs. Conversely, the mRNA expression of *GPX4*, a gene that inhibits ferroptosis, and *PCNA*, a gene that promotes cell proliferation, was significantly downregulated (Figure 2A).

Additionally, the results from protein immunoblotting were consistent with those from qRT-PCR. In comparison to the D280 group, the D580 group’s SWFs showed significantly lower expression of the proteins SLC7A11 and GPX4, which inhibit ferroptosis (Figure 2B,C), and a significantly higher expression of the protein TFRC, which promotes ferroptosis (Figure 2D).

Biochemical analysis revealed that the iron content in SWFs of the D580 group was nearly double that of the D280 group (Figure 2E). Additionally, compared to the D280 group, the D580 group exhibited significantly lower levels of T-SOD, CAT, and GSH, along with significantly higher levels of MDA (Figure 2F).

### 3.3. Effect of Ferroptosis Inhibitor Fer-1 in Attenuating Aging in GCs

**Figure 3:**
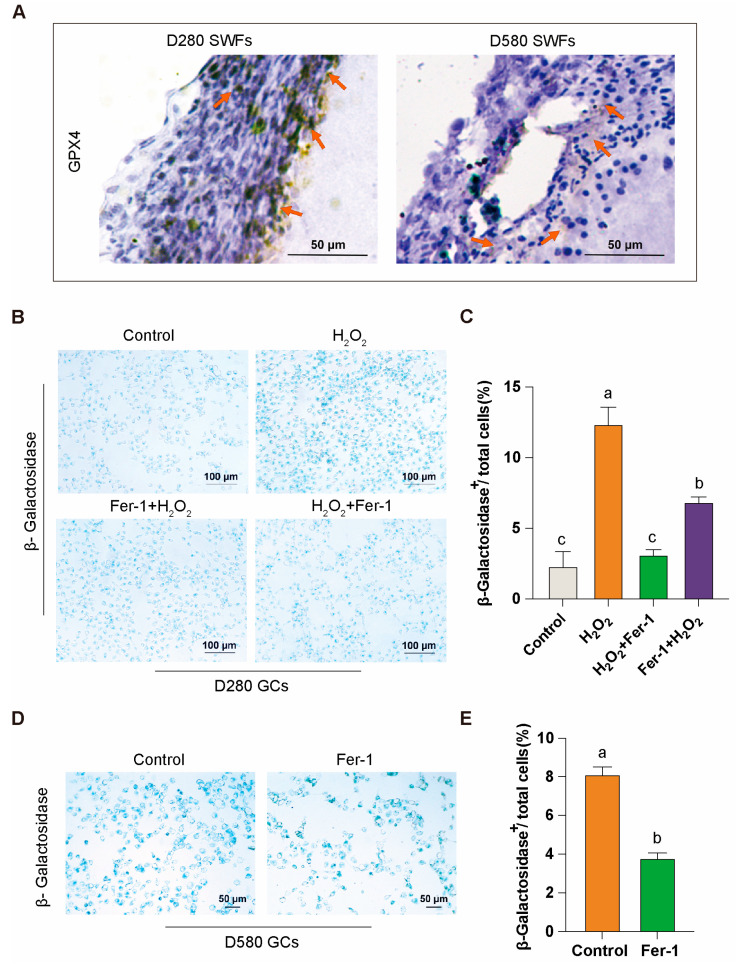
GPX4 expression and Fer-1’s mitigation of granulosa cell aging. (**A**) Immunohistochemical analysis of GPX4 in D280 and D580 chicken SWFs, with orange arrows indicating GPX4 positivity, scale bar: 50 µm. (**B**,**C**) β-galactosidase staining analysis of Fer-1 in the D280 GCs aging model, scale bar: 100 µm. (**D**,**E**) β-galactosidase staining analysis of Fer-1 in D580 GCs, scale bar: 50 µm. Data are presented as means ± SEM (*n* ≥ 3), with different lowercase letters indicating significant differences (*p* < 0.05).

We isolated intact and independent SWFs from D280 and D580 chicken ovarian tissues and conducted immunohistochemical analysis of GPX4. The study revealed that GPX4, a key resistance factor against ferroptosis, is localized in the granulosa layer of the follicles. Additionally, the proportion of GPX4-positive cells in the granulosa layer was reduced in the D580 group compared to the D280 group (Figure 3A). This reduction may be linked to age-related ovarian functional decline.

Subsequently, we directly isolated the granulosa layer from the follicles to obtain GCs for in vitro culture. β-Galactosidase staining showed that in the D280 group, Fer-1 effectively mitigated H_2_O_2_-induced aging in GCs, demonstrating notable preventive and therapeutic effects (Figure 3B,C). In the D580 group, Fer-1 also exhibited some efficacy in alleviating the aging of GCs under natural physiological conditions (Figure 3D,E). These findings suggest that the ferroptosis inhibitor Fer-1 plays a significant role in attenuating GCs aging.

### 3.4. Effect of Ferroptosis Inhibitor Fer-1 on Mitochondria in GCs

**Figure 4:**
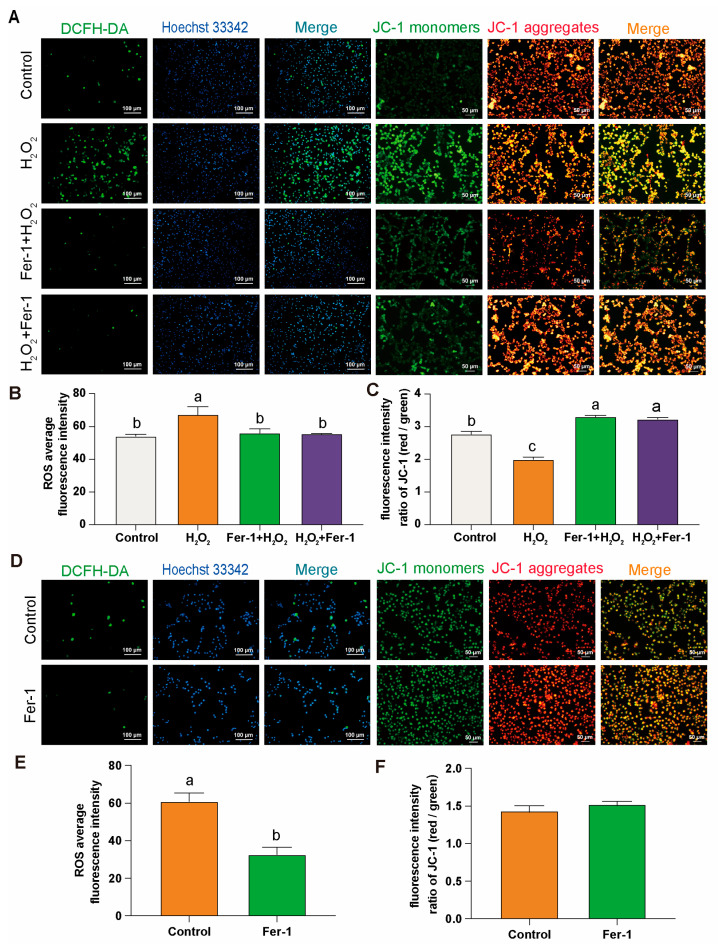
Fer-1 treatment: DCFH-DA and JC-1 staining in GCs. (**A**–**C**) Comparison of DCFH-DA and JC-1 staining in different treatment groups of the D280 GCs aging model with Fer-1. Scale bars: 100 µm or 50 µm, *n* = 3. (**D**–**F**) Comparison of DCFH-DA and JC-1 staining after Fer-1 treatment in D580 GCs, *n* = 3. Scale bars: 100 µm or 50 µm. All data are presented as means ± SEM (*n* ≥ 3), and different lowercase letters indicate significant differences (*p* < 0.05).

After DCFH-DA and JC-1 staining of GCs cultured in vitro from D280 H_2_O_2_ induced and D580 groups, it was found that in the D280 group, the use of Fer-1 for preventive and therapeutic interventions led to a significant decrease in ROS production (Figure 4A,B), and restored ROS levels to a level essentially comparable to that of the control group. Meanwhile, in the Fer-1 intervention group, JC-1 primarily existed in polymer form within the mitochondrial matrix. At this point, there was a significant increase in the red fluorescence intensity within the mitochondria, while the green fluorescence in the cytoplasm notably decreased. The significant increase in the red/green fluorescence ratio indicates a notable improvement in MMP anomalies (Figure 4A,C).

In the D580 group, direct intervention with Fer-1 resulted in a significant decrease in ROS levels in GCs, even lower than physiological levels (Figure 4D,E). Compared to the control group, although the JC-1 staining in the Fer-1 treated group did not show statistical significance, the ratio of red to green fluorescence was still higher than that of the control group (Figure 4D,F).

### 3.5. The Effects of EA Supplementation on Egg Production and Physiology in D580 Chickens

**Figure 5:**
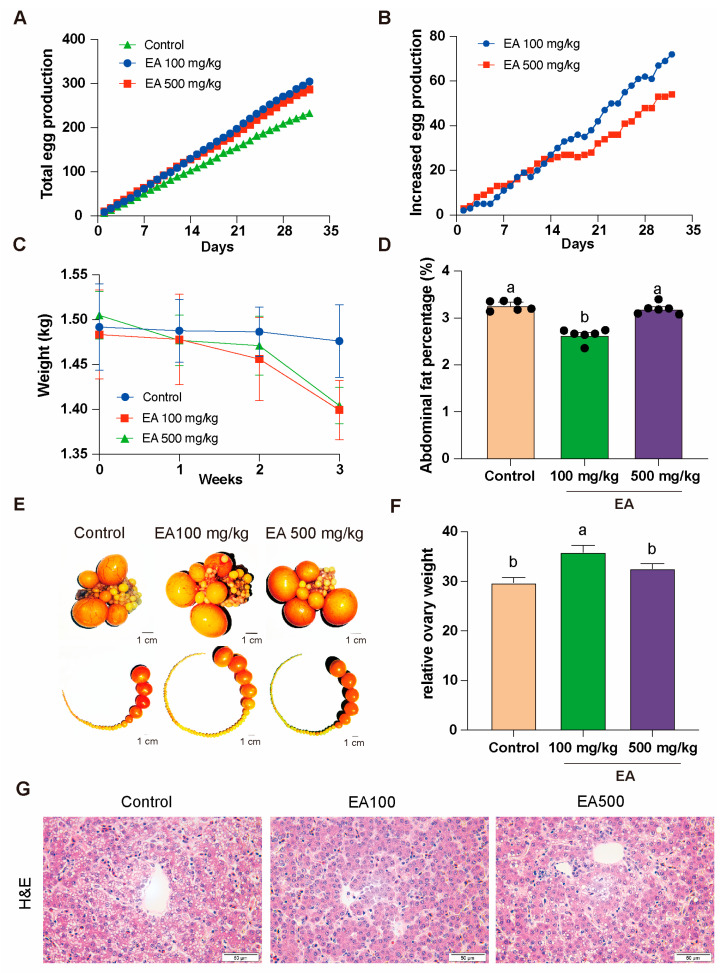
EA effects on egg production and physiological traits in D580 chickens. (**A**) Total egg production in different doses of EA supplementation groups, *n* = 12. (**B**) Daily egg production increment in different doses of EA supplementation groups, *n* = 12. (**C**) Body weight change of chickens in different doses of EA supplementation groups, *n* = 12. (**D**) Abdominal fat rate of chickens in different doses of EA supplementation groups, *n* = 6. (**E**) Distribution of ovaries and ovarian follicles in chickens in different doses of EA supplementation groups, *n* = 12, scale bar: 1 cm. (**F**) Relative weight change of ovaries in chickens in different doses of EA supplementation groups, *n* = 12. (**G**) Hepatic H&E staining in different EA treatment groups, *n* = 3, scale bar: 50 µm. All data are presented as means ± SEM (*n* ≥ 3), and different lowercase letters represent significant differences (*p* < 0.05).

To investigate the effects of EA on the egg-production performance and physiological characteristics of aged chickens, we selected D580 chickens with similar baseline characteristics and randomly divided them into three equally sized groups. Each group received the same basal diet supplemented with different doses of EA (0 mg/kg, 100 mg/kg, and 500 mg/kg) continuously for over 1 month. Following statistical analysis, it was found that chickens fed with EA at low dose (100 mg/kg) and high dose (500 mg/kg) had a higher monthly total egg production, with increases of 32.2% and 26.27%, respectively, compared to the control group (0 mg/kg) (Figure 5A). Notably, around 2 weeks of continuous EA supplementation, the daily egg production increment of the low-dose group began to surpass that of the high-dose group (Figure 5B). Additionally, relative to the control group, chickens fed with both low and high doses of EA exhibited a decrease in body weight (Figure 5C).

Furthermore, we conducted anatomical examinations on the chickens fed with EA at different doses for over a month. The results revealed that the abdominal fat percentage in the low-dose group was significantly lower than that in the control group and the high-dose group and the distribution of ovarian follicles in each group also changed (Figure 5D,E). In the low-dose group, the relative weight of the ovaries was significantly higher than in the other two groups (Figure 5F).

The H&E staining results revealed hepatic steatosis in the control group, characterized by prominent vacuoles within hepatocytes, displaying distinct round or oval shapes with clear boundaries, forming variable-sized transparent areas. Hepatocytes exhibited significant swelling, increased cell volume, and irregular contours. Noticeable fibrosis was observed, presenting a light pink staining, distributed around the hepatic lobules or along the hepatocyte plates. In comparison, the hepatic tissue in the EA 100 mg/kg group exhibited intact cellular architecture without apparent pathological features. In the EA 500 mg/kg group, hepatocytes showed sparse, small vacuolar structures within the cytoplasm, yet the majority maintained normal morphology and orderly arrangement (Figure 5G).

Based on the information in Table 1, it can be concluded that the egg weight in the group of chickens supplemented with 100 mg/kg EA was significantly higher than that of the control group, while the egg weight in the group supplemented with 500 mg/kg EA did not show a significant difference compared to the control group. In terms of eggshell strength, the EA 100 mg/kg group was also significantly higher than the control group, whereas the EA 500 mg/kg group did not show a significant difference from the control group. There were no significant differences in eggshell thickness among the three groups. Additionally, the yolk index and Haugh unit of the EA-supplemented groups were significantly higher than those of the control group. These results indicate that an appropriate amount of EA supplementation (100 mg/kg) can effectively improve egg weight, eggshell strength, and the quality of both yolk and albumen, while a higher dose (500 mg/kg) of EA supplementation did not further enhance these parameters.

### 3.6. The Effect of Different Doses of EA Addition on Different Follicle Populations in D580 Chickens

**Figure 6:**
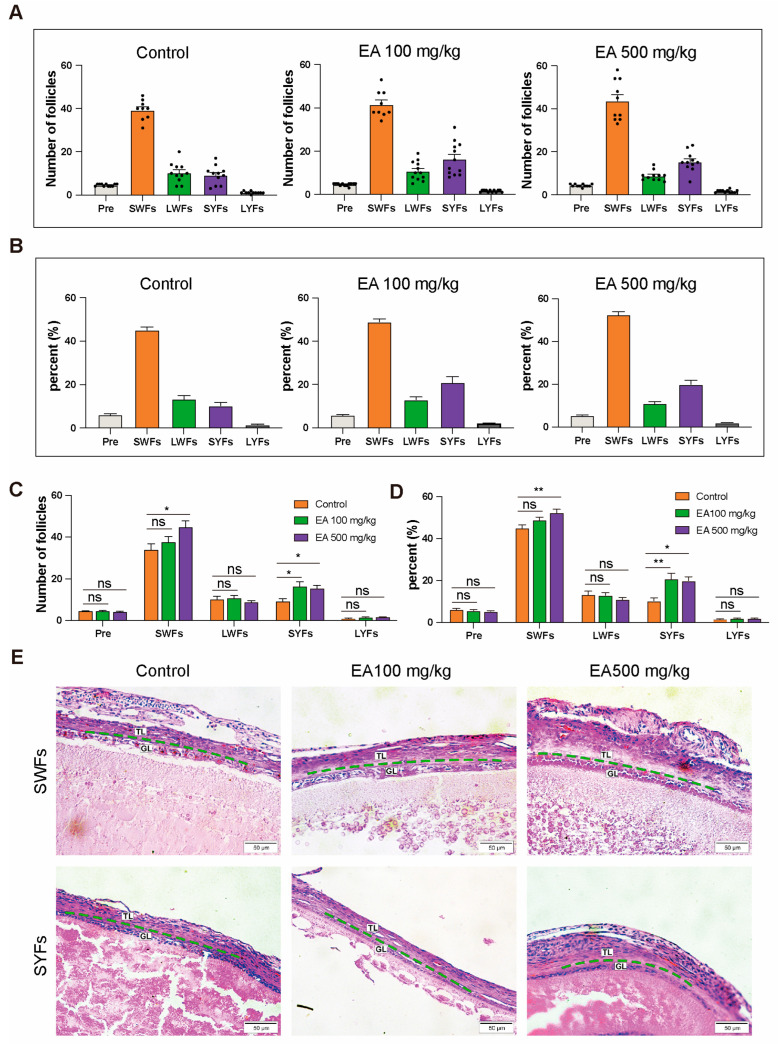
EA supplementation effects on different grades of follicles in D580 chickens. (**A**,**C**) Discrepancies in follicular counts among different EA dosage groups, *n* = 12; (**B**,**D**) Disparities in follicular proportions among different EA dosage groups, *n* = 12; (**E**) H&E Staining of SWFs and SYFs in different EA dosage groups, scale bar: 50 µm, *n* = 3. TL: thecal layer, GL: granulosa layer. Data are presented as means ± SEM (*n* ≥ 3), * *p* < 0.05, ** *p* < 0.01, ns means not significantly.

After dissecting 12 chickens from each group of D580 chickens receiving various doses of EA supplementation upon completing the feeding period, statistical analysis of the data revealed differences in both the quantity and proportion of follicles at different grades among the groups (Figure 6A,B).

Compared with the control group, the EA 100 mg/kg group exhibited a significant increase in the number of SYFs, with a highly significant elevation in their proportion relative to the total follicle count. In the EA 500 mg/kg group, both SWFs and SYFs were significantly more abundant compared to the control group, with SWFs’ proportion exhibiting a highly significant increase, and the proportion of SYFs in the total follicle count also significantly higher than that of the control group. However, there were no significant differences observed in the variation of other follicle types compared to the control group (Figure 6C,D).

The H&E staining results indicated that compared to the control group, the arrangement of cells in the granulosa layer was denser in the 100 and EA 500 mg/kg EA groups, with well-preserved and regular morphology (Figure 6E).

Table 2 shows that the serum biochemical indices of chickens in the EA-treated groups exhibited significantly lower levels of ALT, LDL, TC, and TG compared to the control group, while AST, Glu, and HDL levels showed no significant differences.

### 3.7. Effect of EA on GCs Cycle and Hepatic Lipid Metabolism in Aged Chickens

**Figure 7:**
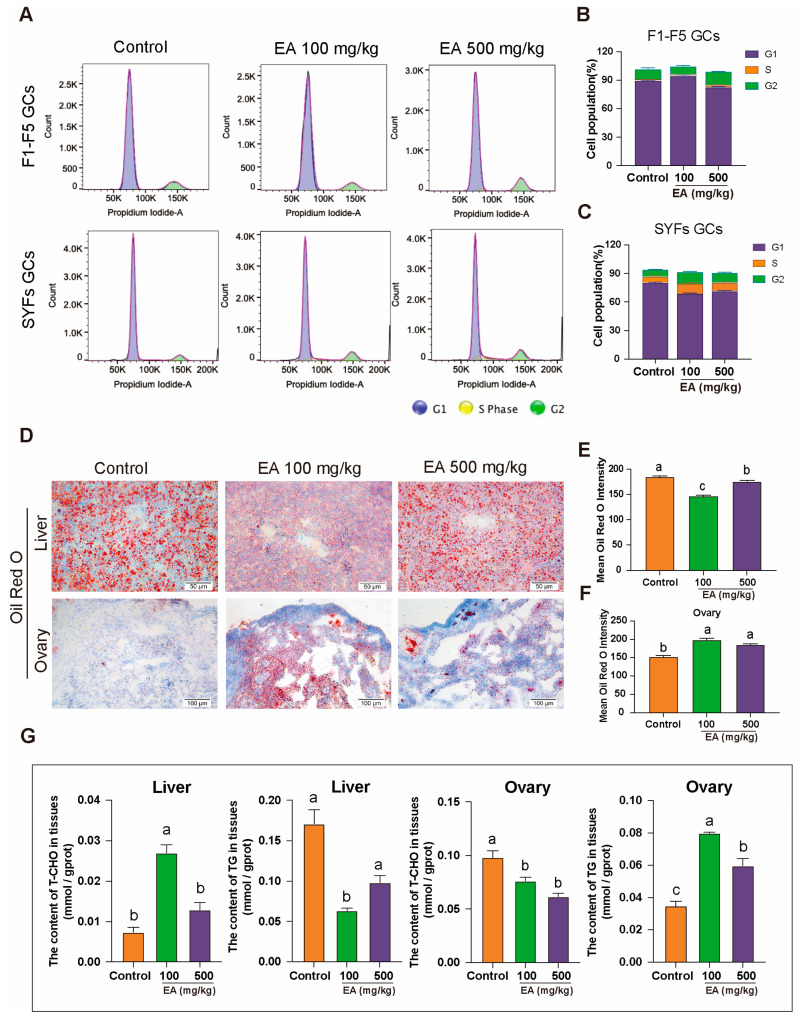
Effect of EA on GCs cycle and hepatic lipid metabolism in D580 chickens. (**A**–**C**) Cell cycle analysis of GCs from F1–F5 and SYFs in D580 chickens under different EA treatments, *n* = 12. (**D**–**F**) Oil Red O staining analysis of liver and ovarian tissues in D580 chickens with different EA supplementation, scale bar: 50 µm, *n* = 3. (**G**) Analysis of T-CHO and TG content in liver and ovarian tissues of D580 chickens with different EA supplementation, *n* = 12. Data are presented as means ± SEM (*n* ≥ 3). Different lowercase letters indicate significant differences (*p* < 0.05).

To further elucidate the impact of GCs status on ovarian aging, three groups of chickens were dissected, and GCs from F1–F5 and SYFs were directly isolated for flow cytometric analysis. The results indicated that in F1–F5, the GCs of aged chickens exhibited G1 phase arrest, preventing the cells from entering the S phase and thereby impairing cell proliferation. In the EA 100 mg/kg group, the cell cycle status was largely consistent with that of the control group. However, although the G1 phase arrest was not effectively alleviated in the EA 500 mg/kg group, there was an extension of the G2 phase (Figure 7A,B). Similarly, in SYFs, the GCs of aged chickens also exhibited G1 phase arrest. In both the EA 100 mg/kg and EA 500 mg/kg treatment groups, the proportion of G1 phase cells entering the S phase increased, and there was an extension of the duration in both the S and G2 phases (Figure 7A,C).

To further investigate whether EA influences egg-laying performance by affecting hepatic lipid metabolism in chickens, Oil Red O staining was performed on liver and ovary tissues from three groups of chickens. The results showed that the Oil Red O staining intensity in the liver tissues of the 100 mg/kg and 500 mg/kg EA groups was significantly lower than that of the control group, with reductions of 33.33% and 16.67%, respectively (Figure 7D–F). Additionally, T-CHO and TG levels were measured in liver and ovary tissues. In the 100 mg/kg EA group, liver T-CHO levels were significantly higher than those in the control group, while TG levels were significantly lower. Conversely, in the ovary tissues, T-CHO levels were significantly lower, and TG levels were significantly higher compared to the control group. In the 500 mg/kg EA group, liver T-CHO and TG levels showed no significant differences from the control group, but the trends in the ovary tissues were similar to those observed in the 100 mg/kg EA group, with T-CHO levels significantly lower and TG levels significantly higher than in the control group (Figure 7E).

### 3.8. EA Alleviates Aging and Oxidative Stress in GCs by Improving Mitochondrial Function

**Figure 8:**
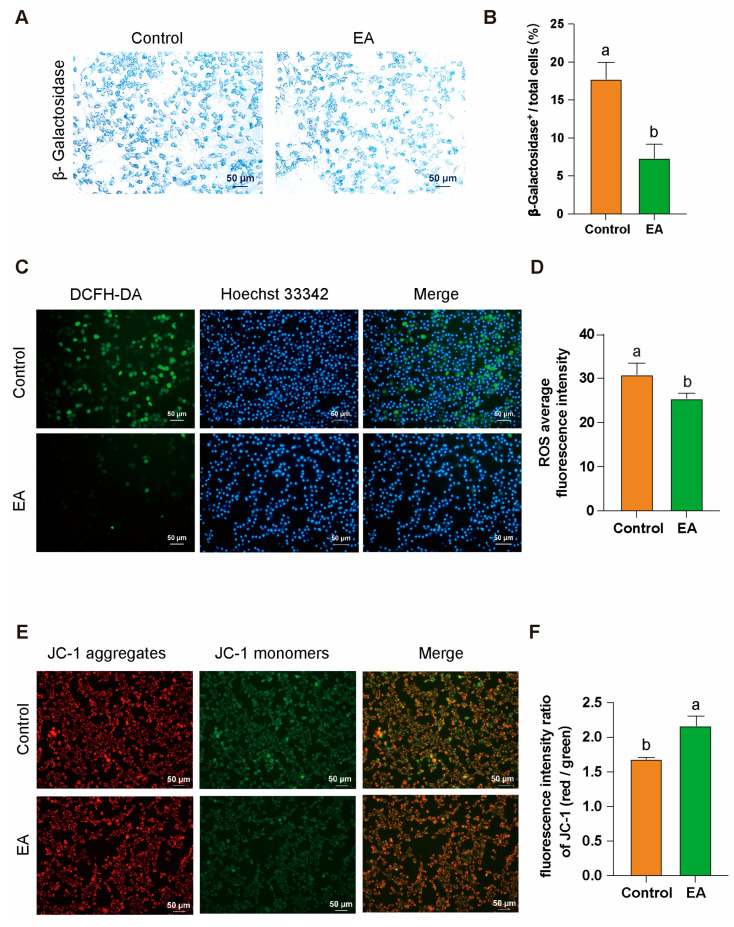
EA alleviates aging and oxidative stress in GCs by improving mitochondrial function. (**A**,**B**) β-Galactosidase staining analysis of GCs after 24 h of in vitro culture with 1 μM EA. Scale bar: 50 µm, *n* = 3. (**C**,**D**) Analysis of ROS production levels in GCs after 24 h of in vitro culture with 1 μM EA, scale bar: 50 µm, *n* = 3. (**E**,**F**) Analysis of MMP abnormalities in GCs after 24 h of in vitro culture with 1 μM EA, scale bar: 50 µm, *n* = 3. All data are presented as means ± SEM (*n* ≥ 3), and different lowercase letters indicate significant differences (*p* < 0.05).

After isolating GCs from D580 chickens, the cells were cultured in vitro with 1 μM EA for 24 h. β-Galactosidase staining analysis revealed that EA effectively alleviated GCs aging under natural physiological conditions, significantly reducing the proportion of β-galactosidase-positive cells (Figure 8A,B). DCFH-DA staining results showed that EA treatment significantly decreased intracellular ROS levels, indicating its potential role in mitigating oxidative stress (Figure 8C,D). Furthermore, JC-1 staining demonstrated a notable improvement in MMP in the EA-treated group (Figure 8E,F). These findings collectively suggest that EA exerts significant anti-aging and cytoprotective effects on GCs.

### 3.9. Effects of EA Supplementation on Mitochondrial Morphology and Ferroptosis-Related Biomarkers

**Figure 9:**
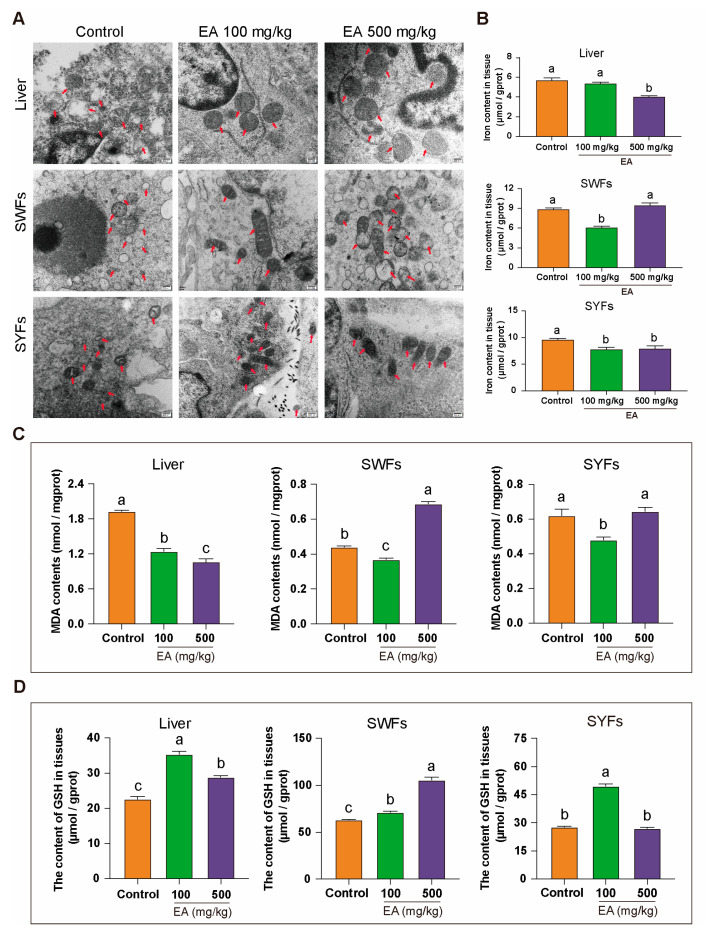
Effects of EA on mitochondrial morphology, tissue iron content, MDA, and GSH levels. (**A**) TEM observation of the effects of different doses of EA supplementation on mitochondrial morphology in liver, SWFs, and SYFs of D580 chickens. Arrow pointing to mitochondria, Scale bar: 200 nm, *n* = 3. (**B**) Analysis of tissue iron content in liver, SWFs, and SYFs of D580 chickens with different doses of EA supplementation, *n* = 12. (**C**) Analysis of tissue MDA levels in liver, SWFs, and SYFs of D580 chickens with different doses of EA supplementation, *n* = 12. (**D**) Analysis of tissue GSH levels in liver, SWFs, and SYFs of D580 chickens with different doses of EA supplementation, *n* = 12. Data are presented as means ± SEM (*n* ≥ 3). Different lowercase letters indicate significant differences (*p* < 0.05).

TEM analysis of liver, SWFs, and SYFs from D580 chickens supplemented with different doses of EA revealed significant differences in mitochondrial morphology. In the control group, mitochondria exhibited severe abnormalities, including extensive shrinkage, blurred or completely disappeared cristae, ruptured and swollen outer membranes, irregular shapes, matrix rarefaction, and the presence of multiple vacuolar structures. Conversely, the groups receiving EA at 100 mg/kg and 500 mg/kg displayed mostly normal mitochondria, with dense and intact cristae, and clearly defined inner and outer membranes. Although some mitochondria still exhibited irregular shapes, the occurrences of mitochondrial shrinkage, cristae loss, and vacuolization were reduced (Figure 9A).

To explore the link between mitochondrial changes and ferroptosis, tissue iron levels were measured. In the liver, iron levels were slightly lower in the EA 100 mg/kg group and significantly lower in the EA 500 mg/kg group compared to controls. In SWFs, iron levels were significantly reduced in the EA 100 mg/kg group but unchanged in the EA 500 mg/kg group, while in SYFs, both EA-treated groups had significantly lower iron levels than the control group (Figure 9B).

This study further examined the levels of MDA and GSH. The results revealed that in the EA 100 mg/kg group, the MDA levels in all tissues were significantly lower compared to the control group. In the EA 500 mg/kg group, the MDA levels in liver tissue significantly decreased, while those in SWFs significantly increased, with no significant difference observed in SYFs (Figure 9C). Additionally, GSH levels were significantly higher in liver and SWFs for both EA-treated groups, with the EA 100 mg/kg group showing a greater increase in the liver, and the EA 500 mg/kg group showing a greater increase in SWFs. In SYFs, GSH levels were higher in the EA 100 mg/kg group but unchanged in the EA 500 mg/kg group (Figure 9D).

### 3.10. Effect of EA on Iron Deposition in Chickens Tissues

To investigate the effect of EA on iron deposition in the liver and ovarian tissues of aged chickens, we conducted Prussian blue staining on the relevant tissues of each group. The results showed that in liver tissues, the control group exhibited prominent Prussian blue deposits in hepatocytes and the central lobular area. In contrast, the EA 100 mg/kg and EA 500 mg/kg groups did not show significant positive iron staining.

**Figure 10:**
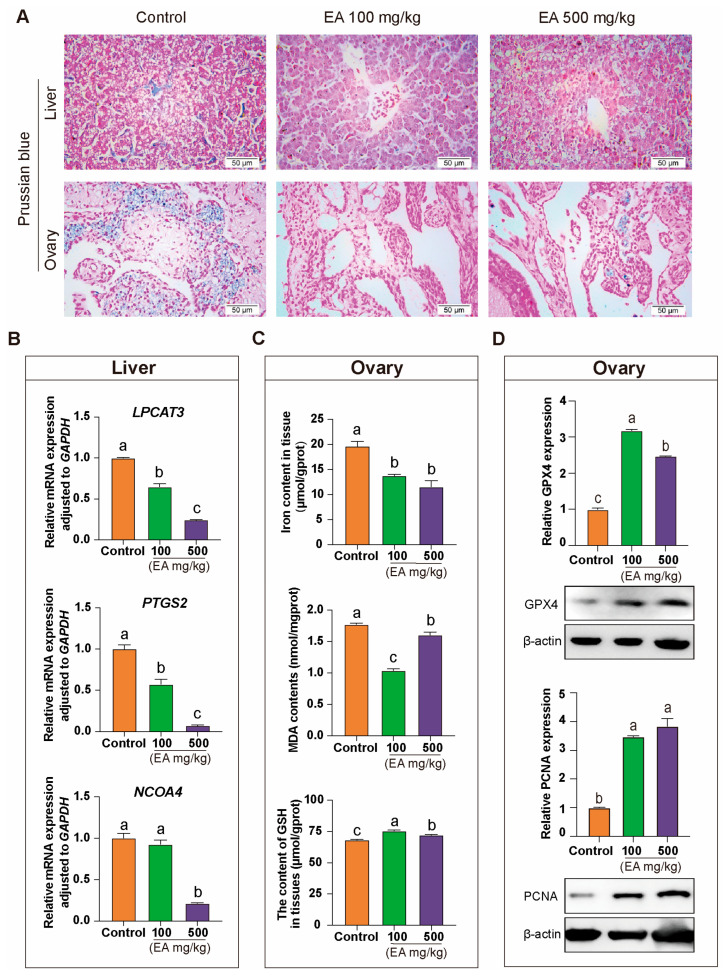
Effects of EA on iron deposition and the expression of iron metabolism-related genes in the liver and ovary tissues of aging chickens. (**A**) Prussian blue staining analysis of liver and ovary tissues from aging chickens in different experimental groups supplemented with EA, scale bar: 50 µm, *n* = 3. (**B**) Differential expression analysis of ferroptosis-promoting genes *LPCAT3*, *PTGS2*, and *NCOA4* in aging chickens supplemented with EA, *n* = 12. (**C**) Analysis of differences in the content of iron, MDA, and GSH in the ovarian tissues of aging chickens from different experimental groups, *n* = 12. (**D**) Protein expression and qRT-PCR analysis of GPX4 and PCNA in the ovaries of aging chickens from different experimental groups, *n* = 12. Data are presented as means ± SEM (*n* ≥ 3), with different lowercase letters indicating significant differences (*p* < 0.05).

In ovarian tissues, we observed a similar pattern. The control group had substantial blue granules in localized regions of the ovaries, while the EA 100 mg/kg group showed almost no positive iron staining. The EA 500 mg/kg group exhibited only a few unevenly distributed positive iron-stained granules (Figure 10A).

In liver tissue, qRT-PCR results indicated that the expression levels of ferroptosis-promoting genes *LPCAT3* and *PTGS2* were significantly lower in the EA-treated groups compared to the control group. Additionally, the expression of the key ferroptosis regulator *NCOA4* was significantly reduced in the EA 500 mg/kg group relative to the other two groups (Figure 10B). In ovarian tissue, the iron content and the ferroptosis product MDA in the control group were significantly higher than in the EA 100 mg/kg and EA 500 mg/kg groups, while GSH levels were significantly lower in the control group compared to the other two groups (Figure 10C). Moreover, the protein expression levels of GPX4 and PCNA in the ovaries were significantly higher in the EA-treated groups than in the control group (Figure 10D).

### 3.11. Effects of EA on Liver, Ovary, and SWFs in Chickens

**Figure 11:**
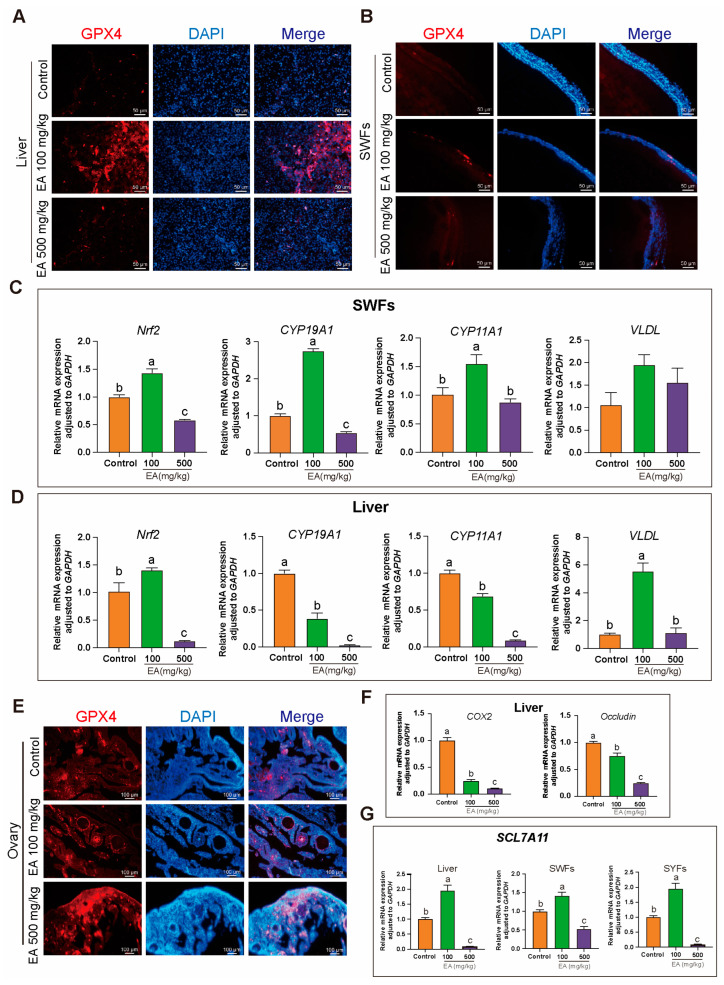
Effects of EA on the expression of antioxidant and related genes in various tissues of aging chickens. (**A**,**B**,**E**) Immunohistochemical analysis of GPX4 in the liver, ovaries, and SWFs of aging chickens supplemented with EA, scale bar: 50 µm, *n* = 3. (**C**,**D**) Expression of *Nrf2*, *CYP19A1*, *CYP11A1*, and *VLDL* genes in the SWFs and liver of aging chickens supplemented with EA, *n* = 12. (**F**) Expression of *COX2* and Occludin in the liver of aging chickens supplemented with EA, *n* = 12. (**G**) Expression trends of *SLC7A11* in the liver, SYFs, and SWFs of aging chickens supplemented with EA, *n* = 12. Data are presented as means ± SEM (*n* ≥ 3), with different lowercase letters indicating significant differences (*p* < 0.05).

Immunohistochemical analysis showed that in the EA 100 mg/kg group, the GPX4 signal in the liver, ovaries, and SWFs of chickens was significantly stronger compared to the other two groups (Figure 11A,B,E). qRT-PCR results indicated that the expression of the antioxidant gene *Nrf2* in the liver and SWFs was significantly upregulated in the EA 100 mg/kg group compared to the control group, whereas it was significantly downregulated in the EA 500 mg/kg group. The expression of *CYP19A1* in the liver was significantly lower in the EA 100 mg/kg group compared to the control group, with the EA 500 mg/kg group also showing a significant decrease. However, in SWFs, the results for the EA 100 mg/kg group were opposite to those in the liver, while the EA 500 mg/kg group followed a similar trend as in the liver. The expression trend of *CYP11A1* was similar to that of *CYP19A1*, but there was no significant difference between the EA 500 mg/kg group and the control group in SWFs. The expression of the *VLDL* gene shows a slightly different pattern. In the liver, the expression of *VLDL* in the EA 100 mg/kg group was significantly higher than in the other two groups. However, there was no significant difference in *VLDL* expression among the groups in the SWFs. (Figure 11C,D). In the liver, the expression of *COX2* and Occludin was significantly lower in both the EA 100 mg/kg and EA 500 mg/kg groups compared to the control group (Figure 11F). Interestingly, the expression trend of the ferroptosis resistance gene *SLC7A11* in the liver, SYFs, and SWFs was also noteworthy. Compared to the control group, the expression of *SLC7A11* was significantly increased in the EA 100 mg/kg group, whereas it was significantly decreased in the EA 500 mg/kg group (Figure 11G).

## 4. Discussion

Poultry-laying performance is closely related to ovarian status, with follicular depletion being a key factor in ovarian functional decline during aging [1]. To explore effective methods to delay ovarian aging, improve the production performance of aging chickens, and extend their productive lifespan, we conducted anatomical observations on the ovaries of Hy-Line White chickens at 280 and 580 days of age under natural physiological conditions. We found that the number and proportion of SWFs in aging chickens’ ovaries were significantly reduced, while other follicle types showed no significant changes. Additionally, the GCs in various follicles of aging chickens were loosely and sparsely arranged. These findings suggest that the decline in ovarian function in aging chickens is closely related to the status of SWFs and GCs.

Natural plant extracts are pivotal in anti-aging and antioxidant research. EA, a natural phenolic compound, is widely present in fruits and nuts such as pomegranates, strawberries, raspberries, and walnuts. Recent studies have shown that EA possesses significant bioactivity and anti-aging potential [26,28]. For instance, EA can markedly improve oxidative stress markers, including lipid peroxidation, CAT, SOD enzyme activities, and GSH concentrations. It mitigates mitochondrial ROS accumulation, prevents MMP abnormalities, reduces mitochondrial swelling, and protects mitochondria from damage. Furthermore, EA supplementation can enhance antioxidant enzyme activities, decrease MDA concentrations in rat ovarian tissues, protect zebrafish embryonic development from DNA oxidative damage, and increase embryo survival rates. However, the effect of EA in mitigating ovarian aging in chickens remains unclear. In this study, EA was supplemented in the diet of aging chickens for over 4 weeks with ad libitum feeding. The results demonstrated that EA significantly improved egg production performance and egg quality, reduced body weight, enhanced liver and ovarian tissue health, optimized follicle distribution, and increased the number and proportion of SYFs and SWFs. At the same time, the EA treatment group showed a tight arrangement of GCs in aging chickens, and the separation of membrane and granulosa layers was significantly improved. To elucidate the mechanisms of EA in delaying ovarian aging and enhancing production performance in chickens, further in-depth investigations were conducted.

Oxidative stress plays a crucial role in aging, with SOD, CAT, and GSH forming a key antioxidant system [30]. SOD converts superoxide anions (O_2_^−^) into H_2_O_2_, serving as the first line of defense, while CAT decomposes H_2_O_2_ into water and oxygen, reducing its toxicity. GSH, as the primary non-enzymatic antioxidant, not only directly scavenges free radicals but also serves as a substrate for glutathione peroxidase, further mitigating oxidative stress [17]. With aging, the activities and levels of these antioxidants decline significantly, leading to increased oxidative stress, cellular damage, and the promotion of aging. Simultaneously, GSH also plays a critical regulatory role in preventing ferroptosis, a type of cell death driven by iron-dependent lipid peroxidation and closely associated with aging. During lipid peroxidation, large amounts of MDA are produced, serving as a key marker of ferroptosis [10,16]. Elevated MDA levels indicate increased lipid peroxidation and cellular damage. GSH, as a cofactor of GPX4, effectively reduces lipid peroxides, preventing their accumulation and lowering MDA levels, thereby inhibiting ferroptosis. In this study, the supplementation of EA significantly increased the levels of SOD, CAT, and GSH in the ovaries and liver tissues of aged chickens and inhibited the production of MDA. Therefore, we preliminarily conclude that EA may delay ovarian aging in chickens by modulating the ferroptosis pathway [26].

Complete follicles are composed of oocytes, GCs, and TCs. GCs play a critical role in follicle development by secreting factors and steroid hormones [2,31]. Cellular senescence is defined as a permanent cell cycle arrest with resistance to growth factors and other signals that induce cell proliferation, representing an irreversible state [32]. In vitro studies, the expression of the proliferation marker gene *PCNA* in D580 SWFs of naturally aging laying chickens was significantly downregulated. Conversely, in vivo studies, the EA treatment group showed a significant upregulation of PCNA expression. Moreover, EA supplementation promoted the transition of cells from the G1 phase to the S phase and extended the duration of the S and G2 phases. The upregulation of PCNA and the promotion of G1/S transition in granulosa cells may be directly associated with increased follicular development and laying capacity. The upregulation of PCNA indicates active cell proliferation, and the transition from G1 to S phase marks the entry of granulosa cells into the proliferation stage, supporting follicular growth and maturation. By enhancing granulosa cell proliferation, follicles acquire more supporting cells, promoting their development and maturation, ultimately improving ovarian laying capacity. This indicates that EA can alleviate the aging of GCs by promoting cell proliferation.

H_2_O_2_, a natural inducer of oxidative stress, is most commonly used as a model for oxidative stress-induced premature senescence [33]. In this study, H_2_O_2_ was used to induce senescence in an in vitro model of GCs, and subsequent treatment with the ferroptosis inhibitor Fer-1 was administered. We found that Fer-1 effectively alleviated H_2_O_2_-induced GCs senescence, reduced abnormal accumulation of reactive ROS, and improved mitochondrial membrane potential abnormalities. Moreover, Fer-1 exerted similar effects in naturally aging GCs from D580 chickens. The mechanism by which Fer-1 alleviates senescence is very similar to that of EA. This further indicates that EA may alleviate ovarian aging in chickens by inhibiting ferroptosis.

Mitochondria are the primary sites for iron metabolism and the Fenton reaction during ferroptosis. In this process, the Fenton reaction generates highly reactive hydroxyl radicals (•OH), initiating lipid peroxidation. This leads to a significant reduction in MMP, increased membrane permeability, and the accumulation of large amounts of lipid peroxidation-derived ROS, further promoting lipid peroxidation and cellular damage. Morphologically, ferroptosis is characterized by reduced mitochondrial volume, decreased or missing cristae, and thickened outer membranes [34]. Key genes such as *LPCAT3*, *NCOA4*, *PTGS2*, *COX2*, *TFRC*, and *GPX4* also play critical roles in ferroptosis [35]. *LPCAT3* promotes lipid peroxidation through phospholipid remodeling, *NCOA4* increases intracellular iron content via ferritinophagy, *PTGS2/COX2* upregulation facilitates the formation of lipid peroxides, *TFRC* enhances intracellular iron accumulation, and *GPX4* inhibits ferroptosis by reducing lipid peroxides [36,37]. These genes collectively regulate oxidative stress levels in ferroptosis, forming a complex regulatory network. Transmission electron microscopy of aging chicken liver, SWFs, and SYFs mitochondria in various study groups revealed that mitochondrial morphology in the control and high-dose EA groups resembled the typical features observed during ferroptosis. In contrast, significant morphological improvements were observed in the low-dose EA group. In vitro studies on GCs showed that EA significantly alleviates GCs aging, restores abnormal MMP, and reduces ROS accumulation in aging chicken GCs. Compared to the D280 group under natural physiological conditions, the D580 group showed significantly upregulated expression of *LPCAT3*, *NCOA4*, *PTGS2*, *COX2*, and *TFRC*, while *GPX4* was significantly downregulated in SWFs. Additionally, in aging chickens’ SWFs, the levels of SOD, CAT, and GSH are significantly decreased, while the level of MDA is significantly increased. This indicates that ferroptosis and oxidative stress pathways jointly regulate ovarian aging in chickens. However, the specific mechanisms by which EA alleviates chicken ovarian aging through oxidative stress and ferroptosis pathways remain to be explored.

The synthesis of GSH is regulated by various factors, including the activation of the Nrf2 signaling pathway. Nrf2 is a transcription factor that can regulate the expression of multiple antioxidant enzymes and antioxidants. Under ferroptotic conditions, activation of the Nrf2 pathway can promote GSH synthesis, thereby enhancing cellular antioxidant capacity. According to qRT-PCR results, EA can upregulate the *Nrf2* gene and regulate GSH content and GPX4 expression, thereby enhancing ovarian antioxidant defenses and liver function, and protecting cells from oxidative damage [38]. Additionally, *Nrf2* indirectly influences the expression of *CYP19A1* and *CYP11A1*, enzymes involved in steroid hormone synthesis [39]. The expression of CYP19A1 and CYP11A1 in the liver was significantly altered in the 100 mg/kg EA group. qRT-PCR showed that CYP19A1 was significantly lower in the liver of the 100 mg/kg EA group compared to the control, with a similar decline observed in the 500 mg/kg EA group. *CYP19A1* regulates lipid metabolism by converting androgens into estrogens, which reduces LDL and increases HDL levels, thereby influencing TG and very low-density lipoprotein (VLDL) levels [40].

In SWFs, the 100 mg/kg EA group showed an opposite trend, with a significant increase in CYP19A1 expression, while the 500 mg/kg EA group exhibited a similar decrease as in the liver. The expression of CYP11A1 followed a similar pattern to CYP11, with a significant decrease in the liver. *CYP11A1* converts cholesterol into pregnenolone, maintaining cholesterol homeostasis and impacting lipid metabolism through the regulation of steroid hormone levels. In summary, these two enzymes significantly influence the balance and metabolism of TG, T-CHO, and VLDL by modulating estrogen and other hormone levels, thereby affecting body weight and yolk deposition in chickens [41]. Immunohistochemistry results also show that the 100 mg/kg EA group exhibits strong GPX4 signals in the liver, ovaries, and small white follicles. EA may regulate GPX4 and GSH to collectively counteract ferroptosis, thereby alleviating ovarian aging in chickens.

## 5. Conclusions

**Figure 12:**
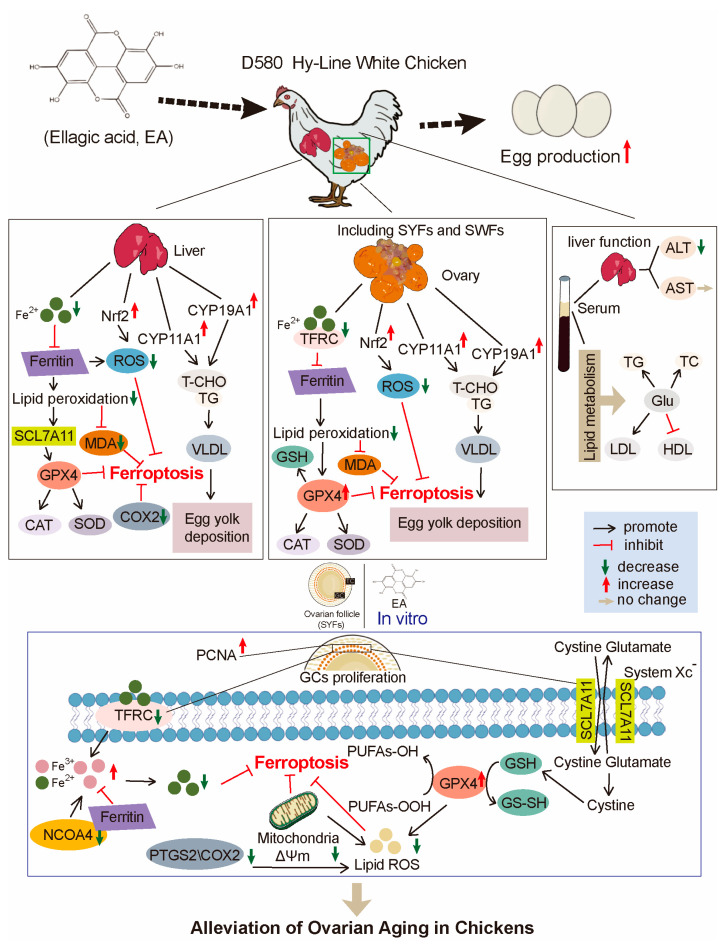
Schematic model illustrating the effect of EA in regulating ferroptosis, oxidative stress, and lipid metabolism in chickens.

This study demonstrated that EA effectively alleviates H_2_O_2_-induced aging in GCs by promoting GPX4 expression, which reduces oxidative stress, inhibited ferroptosis, and decreased ROS levels while regulating mitochondrial function. Additionally, EA significantly enhances the mRNA and protein expression of the cell proliferation-related gene *PCNA*, alleviating G1 phase arrest in aging GCs. In vivo, EA increased the number, proportion, and relative weight of SYFs in aging chickens, upregulated yolk deposition-related genes, and improved serum antioxidant levels, which contributed to reduced fat accumulation. Ultimately, these effects suggested that EA effectively delayed ovarian aging in chickens by regulating antioxidant capacity and ferroptosis mechanisms, thereby enhancing the production performance of low-yield laying chickens (Figure 12).

In conclusion, EA has shown significant effects in delaying ovarian aging in laying chickens, providing a new strategy for extending the productive lifespan of poultry and enhancing their production efficiency.

## Figures and Tables

**Table 1:** Effects of EA supplementation on physical quality of eggs.

Item	Control	EA 100 mg/kg	EA 500 mg/kg	*p*-Value
Egg weight, g	62.87 ± 0.53 ^b^	67.36 ± 1.19 ^a^	64.86 ± 0.83 ^ab^	0.027
Shell strength, kg/cm^2^	2.57 ± 0.15 ^b^	4.25 ± 0.56 ^a^	3.34 ± 0.25 ^ab^	0.047
Shell thickness, mm	0.38 ± 0.01	0.41 ± 0.01	0.41 ± 0.01	0.092
Yolk index	0.37 ± 0.01 ^b^	0.43 ± 0.01 ^a^	0.42 ± 0.02 ^a^	0.006
Haugh Unit	71.98 ± 2.90 ^b^	85.06 ± 2.16 ^a^	78.64 ± 1.41 ^a^	0.004

^a,b^ Means within a row with different superscripts differ significantly (*p* < 0.05).

**Table 2:** Biochemical parameters of chicken serum.

Item	Control	EA 100 mg/kg	EA 500 mg/kg	*p*-Value
ALT, U/L	15.80 ± 2.94 ^a^	8.27 ± 1.04 ^b^	8.30 ± 1.18 ^b^	0.0161
AST, U/L	199.84 ± 7.01	196.11 ± 9.27	197.36 ± 9.57	0.9718
Glu, mmol/L	12.28 ± 0.35	11.35 ± 0.34	11.58 ± 0.27	0.1376
HDL, mmol/L	0.32 ± 0.03	0.34 ± 0.02	0.34 ± 0.02	0.7886
LDL, mmol/L	1.34 ± 0.11 ^a^	0.77 ± 0.12 ^b^	0.77 ± 0.09 ^b^	0.0066
TC, mmol/L	3.24 ± 0.24 ^a^	2.13 ± 0.17 ^b^	2.44 ± 0.19 ^b^	0.0019
TG, mmol/L	17.36 ± 0.57 ^a^	13.73 ± 0.55 ^b^	13.75 ± 0.95 ^b^	0.0004

^a,b^ Means within a row with different superscripts differ significantly (*p* < 0.05). ALT Alanine aminotransferase, AST Aspartate aminotransferase, Glu Glucose, HDL High-density lipoprotein, LDL Low-density lipoprotein, TC Total cholesterol, TG Triglycerides.

## Data Availability

All data analyzed are contained within the article.

## Supplementary Materials

The following supporting information can be downloaded at: https://www.mdpi.com/article/10.3390/antiox14050614/s1, Table S1: Primers for qPCR analysis.
