# Supplementary material for: Attenuating Effect of a Polyphenol Ellagic Acid on Ovarian Aging by Inhibiting the Ferroptosis Pathway in Low-Yield Laying Chickens
Source: Antioxidants (Basel). 2025 May 21;14(5):614. doi: 10.3390/antiox14050614 (PMC12109511; doi:10.3390/antiox14050614)
Supplement: Supplementary file 1 [file antioxidants-14-00614-s001.zip › antioxidants-3616667-supplementary.pdf]

Supplementary Table S1. Primers for qPCR analysis.

Gene name	Accession number	Primer sequence (5'-3')	Product size (bp)
CYP11A1	NM_001001756.2	F: GGTGGCATACCGTGACTACC<br>R: ACAAAGTCCTGGCTCACCTG	159
CYP19A1	NM_001001761	F: CCTCTGCTGGAGATGGTTTT<br>R: GCTGATCCACTTTAGTCACTCTGA	68
NCOA4	NM_001006495	F: CACTCCTACAATAACGAACT<br>R: TCAGAACAGAAGACTCCTC	94
TFRC	NM_205256.2	F: CAGAGGCAGCACCAAGAACCAAG<br>R:TTTCAGCCCGTAGTTCACATAGACAG	167
LPCAT3	XM_416516.6	F: ACTTCACAGCCACAGAGCACTATG<br>R:GCCAGACCAATCAACTTGAGAGTCA	79
GPX4	AF498316.2	F: GCTACGGGGTGAAGTTCGACATG<br>R: TGAAGTTCCACTTGATGGCATTCCC	150
PTGS2	NM_001167719.2	F:ACACATTCCAGCAGTTCCTCTACAA<br>R: TTCCCACCAGCAACCCTACCA	109
PCNA	NM_204170.2	F: GGGCGTCAACCTAAACAGCA<br>R: AGCCAACGTATCCGCATTGT	97
Occludin	NM_205128.1	F: CTCTGGGAAGGGCTGAGGT<br>R: GCCTTCCCAAAAAGCCCTGA	170
Nrf2	NM_001030756.1	F: CTGCTAGTGGATGGCGAGAC<br>R: CTCCGAGTTCTCCCCGAAAG	132
COX2	NM_001167719.1	F: CTGCTCCCTCCCATGTCAGA<br>R: CACGTGAAGAATTCCGGTGTT	123
VLDL	NM_205229.1	F: ATGGCCAGGATCGTAGACTT<br>R: TCATTTATCTGAGGAGCAGG	292
GAPDH	NM_204305.2	F: CAGAACATCATCCCAGCGTCCAC<br>R: GGCAGGTCAGGTCAACAACAGAG	133
